# Preemptive Striking in Individual and Group Conflict

**DOI:** 10.1371/journal.pone.0154859

**Published:** 2016-05-05

**Authors:** Nobuhiro Mifune, Yoichi Hizen, Yoshio Kamijo, Yoshitaka Okano

**Affiliations:** School of Economics and Management, Kochi University of Technology, Kochi-city, Kochi, Japan; Middlesex University London, UNITED KINGDOM

## Abstract

In this study, we conducted a laboratory experiment to assess preemptive striking by and towards individuals or groups. In the framework of a preemptive strike game, we set the following four conditions: one person faced another person, one person faced a three-person group, a three-person group faced an individual, and a three-person group faced another three-person group. Previous studies have revealed that greed is activated when participants belong to a group, while fear is activated when participants interact with a group, and further, that attacking behaviors in the preemptive strike game are driven by fear. These observations led to a hypothesis that high attack rates would be realized when participants interact with a group, regardless of whether the participants make decisions as individuals or a group. The results of our experiment, however, rejected this hypothesis. Among the four conditions, the attack rate was highest when a three-person group faced an individual. As possible reasons for our observation, we discuss the potential threat stemming from the imbalance in the effectiveness of attack between individuals and groups, and the (incorrect) belief by groups that single individuals would be more likely to attack out of fear.

## Introduction

“So that in the nature of man, we find three principal causes of quarrel. First, Competitions; Secondly, Diffidence; Thirdly, Glory”

(Thomas Hobbes, 1651 “Leviathan” [1]).

Intergroup conflict such as warfare has been ubiquitous in our social world for a million years [2, 3], and in prehistoric times was even more widespread than at present [4]. Many researchers have examined the mechanism or cause of conflicts in various fields of social sciences, including sociology [2], political science [5], economics [6], and so on. Although there are several points of view, fear or diffidence [1] may play a key role in explaining intergroup conflicts.

Preemptive striking is associated with war, and is likely to be driven by fear. People sometimes advocate, support, or even engage in preemptive strikes if they feel fear or, in other words, perceive that the opponent will/may attack them [7]. As a good example of support for fear-based preemptive strikes, the Administration of then-President George W. Bush clearly stated the importance of the pre-emption in their National Security Strategy, and a substantial majority of American people approved of the doctrine of Bush, which included the notion of preemptive strike against Iraq in the wake of the 9/11 attack [8]. Fear could result in preemptive strikes, and then, warfare.

This fear-based preemptive strike can be measured in the preemptive strike (PS) game [9]. The PS game was invented to measure people’s infliction of an attack on another party where no explicit incentives to harm the opponent exist. In this game, participants have some portion of initial money (e.g., 500 yen) and decide whether to click a button on the PC screen (i.e., commit a preemptive strike) or not. If both parties refrain from clicking the button, they keep their initial money. If one party clicks the button, it inflicts a large cost to the other party (e.g., 400 yen) while it costs a smaller amount to that party (e.g., 100 yen). If both parties click the button, a first-come, first-served principle is followed; that is, only the earlier strike is effective. In this game structure, clicking the button is the optimal behavior for each party only if the party expects the other party to click the button, although the party has no reason to expect this of the other party’s behavior. In fact, if both parties refrain from clicking the button, they can achieve the largest payoffs (i.e., 500 yen). In this sense, such an expectation is regarded as fear.

Simunovic et al. [9] revealed that participants in the PS game attack their opponents because of fear, not out of spite (i.e., motivation to maximize their own gain relative to their opponents even if their attack leads to a decline in their own absolute reward; note that spite has often been suggested to motivate behavior in a number economic games [10, 11]). In their study, some participants faced opponents who did not have the option to click the button. Hence, they would not feel fear but could have been motivated by spite. In this situation, participants had rarely attacked (i.e., only one out of 26 participants). On the other hand, participants who could attack each other showed a nearly 50% attack rate. These results suggest that people commit preemptive strikes when they feel fear toward the other party in the PS game, but that attacks are not based on spite.

The aim of this study was to investigate preemptive strikes by and against groups. Many studies, mostly using the prisoner’s dilemma (PD) game, support the prediction that more severe competitiveness will emerge in intergroup interaction as compared to interindividual interaction (for reviews, see references [12, 13]). Groups tend to make competitive or non-cooperative choices rather than cooperative choices when they interact with other groups as opposed to when they are with other individuals, which is called the interindividual-intergroup discontinuity effect [14].

Two possible explanations for the discontinuity effect exist. One is based on fear, wherein people have the naïve intuition that groups are more competitive, aggressive, and outrageous than individuals [15]. This intuition or expectation induces people to make defensive non-cooperative or competitive choices when they interact with groups rather than individuals [14, 16]. The other explanation for the discontinuity effect is based on greed. In the context of the PD game and public goods game, greed is defined as the temptation to free ride on the other party’s cooperation [17, 18]. More generally, greed can be defined as the motivation to pursue one’s own payoffs on the basis of the expectation that the other party will cooperate. In a group, people tend to feel anonymous [19], support shared self-interest [20, 21], and conform to the norm of ingroup favoritism [22]. Therefore, belonging to a group elevates greed. Several previous studies revealed that the discontinuity effect occurs because of both fear and greed [12, 13].

One possible way to examine whether the discontinuity effect occurs because of fear, greed, or both is to manipulate the number of own-group members and the number of the opponent group's members, and set four conditions [23, 24]: one participant interacts with another individual participant (1 vs. 1 condition), one participant interacts with a three-person group (1 vs. 3 condition), a three-person group interacts with an individual participant (3 vs. 1 condition), and a three-person group interacts with another three-person group (3 vs. 3 condition). In general, the degree of fear should increase as the number of the opponent group’s members increases, irrespective of the number of own-group members. On the other hand, the degree of greed should increase as the number of own-group members increases, irrespective of the number of the opponent group’s members. Previous studies using the PD game have revealed that the non-cooperation rate in the 3 vs. 3 condition is higher than that in the 1 vs. 1 condition, and the non-cooperation rates in the 1 vs. 3 and 3 vs. 1 conditions are between those of the 3 vs. 3 and 1 vs. 1 conditions [24].

The current study investigated how the attack rate in the PS game changes according to the number of own-group members and the number of the opponent group’s members. Because Simunovic et al. [9] revealed that only fear operates in the PS game, we expected that participants who interact with a group would attack more frequently than when they face an individual participant, whereas the number of own-group members would not affect the attack rate. Concretely speaking, it was expected that a significant difference in aggression rate would be observed between the 1 vs. 1 and 1 vs. 3 conditions, and also between the 3 vs. 1 and 3 vs. 3 conditions.

## Materials and Methods

### Ethics statement

The study was approved by the research ethics committee of Kochi University of Technology. All participants signed the written informed consent at the beginning of the experiment.

### Design

The PS game is played between two parties. We set four between-subjects conditions, 1 vs. 1 (one participant plays against another participant), 1 vs. 3 (one participant plays against a group of three participants), 3 vs. 1 (a group of three participants plays against a participant), and 3 vs. 3 (a group of three participants plays against another group of three participants).

### Participants

Three-hundred ninety-nine undergraduate and graduate students participated in our experiment (264 men, 135 women). The mean age was 19.5 (SD = 1.52). Monetary rewards were emphasized as incentive for their participation.

One participant did not show up in the 3 vs. 1 condition; therefore, we have one observation for a 2 vs. 1 condition. Findings of statistical significance did not change regardless of whether this observation was included or excluded. In our main analysis, therefore, we included it in the 3 vs.1 condition. The sample size used for our statistical analysis was 200 in total, 50 for each condition.

### The payoff structure of the preemptive strike game

All participants were given 500 yen as their show-up fee and an additional 500 yen for the PS game. If both parties refrained from pushing the button on their PC screens, each participant ended up with the initial endowments. Note that in the case of the three-person group, 500 yen was given to each member. The attacking party, who pushed the button earlier than the other party, paid 100 yen per person as a cost, while the attacked party, who pushed the button later or did not push the button, paid 400 yen per person. Hence, the largest payoff in the PS game was 500 yen, followed by 400 yen, and 100 yen for each person.

### Procedure

Participants arrived at the reception desk by the starting time of each session. Because we initiated reception in advance of the starting time, some participants could see other participants at the reception desk but others could not.

The laboratory was located at a distance from the reception desk and had two large and two small cubicles. Each three-person group (and only in the 1 vs. 1 condition, one participant) entered a large cubicle, while each small cubicle was occupied by only one participant. Because large cubicles were soundproof, the three participants in each group could talk secretly. Which cubicle each participant entered was determined by a lottery distributed at the reception desk, and participants were invited from the reception desk to the laboratory one by one. Thus, participants could not identify which participants they saw at the reception desk had entered each cubicle unless they happened to get together in the same large cubicle as the members of the same three-person group. After this, participants took their seats in each cubicle and signed the written informed consent, and an instruction sheet was distributed to each participant.

The computer-based PS game developed in Visual Studio 2010 was conducted. In the case of three-person groups, only one PC was provided in each large cubicle and one of the three participants operated the PC. The game contained two phases: a rehearsal phase and a real phase. In the rehearsal phase, each individual participant and each three-person group independently experienced each of the three possible outcomes of the PS game: win, loss, and withdrawal. This was not an actual play with other participants; the predetermined result was shown on their PC screens regardless of how quickly they pushed the attack button. After all participants finished the rehearsal phase, 5 minutes were given to participants to determine whether to push the button. In the case of three-person groups, participants were asked to discuss amongst themselves and decide whether to push the button. A 5-second countdown was then followed by the 30-second real phase of the PS game.

After the game, participants answered post-experimental questionnaires consisting of demographic items and were paid rewards according to the result of the PS game. The experiment took about 30 minutes.

## Results

First, in order to examine whether the 5-minute discussion was enough for three-person groups to achieve agreement, we conducted a one-way ANOVA on the time spent to push the attack button after the 30-second real phase started. If the 5 minutes were enough for three-person groups, the reaction time would show no significant difference between three-person groups and individuals. For individuals and groups who actually pushed the button in the 30 seconds, the mean time spent to push the button was 616 milliseconds (standard deviation = 294) in the 1 vs. 1 condition, 1175 ms (SD = 2961) in the 1 vs. 3 condition (for one individual in this condition, the time score was not recorded because of a programming error), 431 ms (SD = 211) in the 3 vs. 1 condition, and 367 ms (SD = 212) in the 3 vs. 3 condition. There were no significant differences among the four conditions (F(3, 91) = 1.64, p = .185). In the 1 vs. 3 condition, one participant pushed the button at 12611 ms. If this observation was eliminated, the mean reaction time in the 1 vs. 3 condition was 461 ms (SD = 300). Simunovic et al. [9] reported that almost all participants who pushed the button did so within the first second of the game. Therefore, the reaction time data in the current experiment are consistent with the previous study [9].

**Fig 1:**
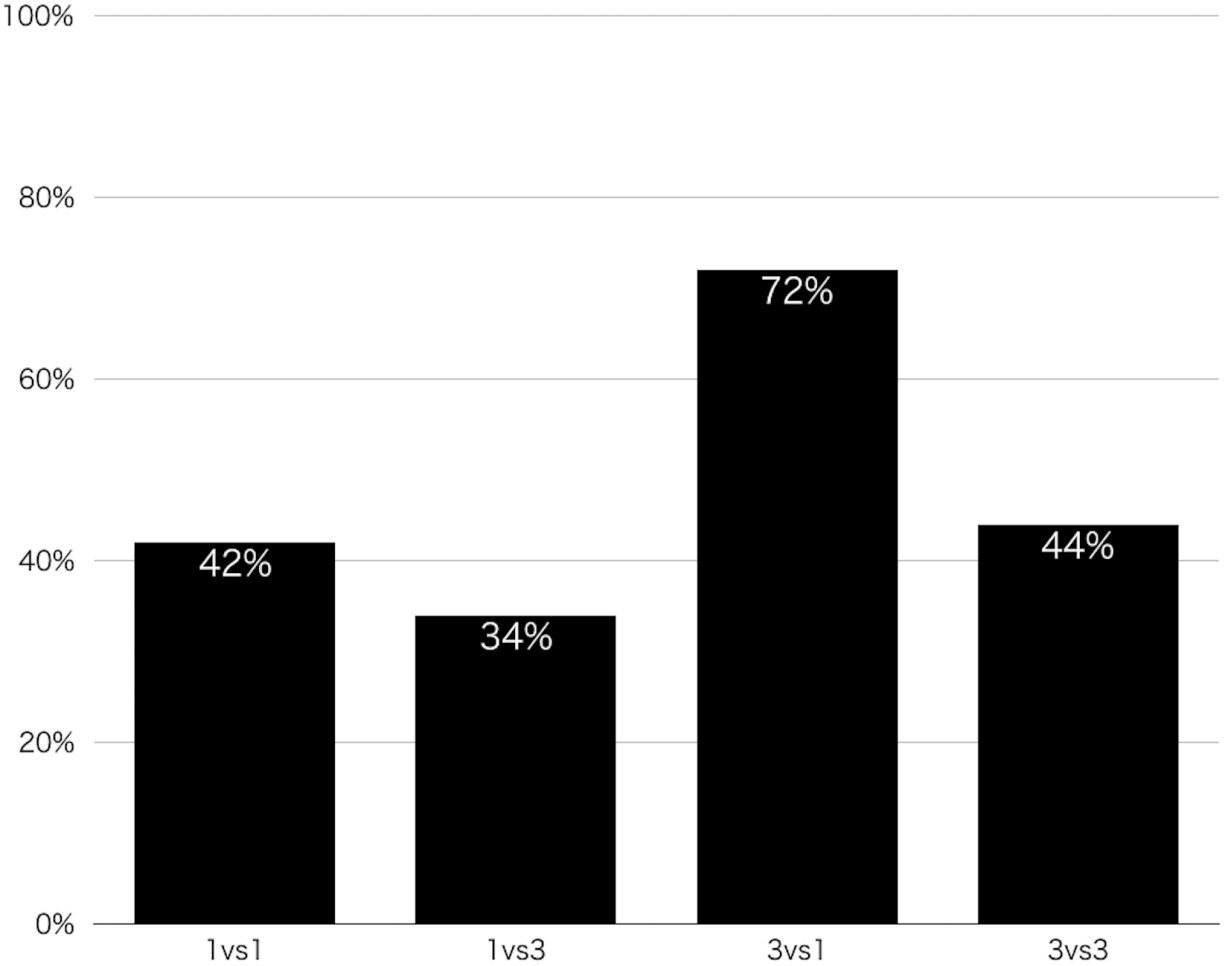
Attack rate in the four conditions.

We conducted Pearson's chi-square tests to examine whether the frequency of attacks was dependent on the four conditions. The ratio of parties who pushed the button showed a significant difference among the four conditions as a whole (χ^2^ (3, N = 200) = 16.51, p < .001, Fig 1), although no significant difference was observed between the 1 vs. 1 and 3 vs. 3 conditions (χ^2^ (1, N = 100) = 0.04, p = .840). This provides evidence that the discontinuity effect may not occur in the PS game. In addition, the attack rate in the 1 vs. 3 condition did not significantly differ from that of the 1 vs. 1 condition (χ^2^ (1, N = 100) = 0.68, p = .410) or that of the 3 vs. 3 condition (χ^2^ (1, N = 100) = 1.05, p = .305). The attack rate of the 3 vs. 1 condition was approximately twice as high as those of the 1 vs. 1 (χ^2^ (1, N = 100) = 9.18, p = .002), 3 vs. 1 (χ^2^ (1, N = 100) = 14.49, p < .001), and 3 vs. 3 (χ^2^ (1, N = 100) = 8.05, p = .005) conditions.

## Discussion

We examined preemptive striking by and against individuals and groups. The current study showed unpredicted results. About 40% of participants engaged in preemptive striking in interindividual interaction (i.e., 1 vs. 1 condition), which is almost the same as in the previous study [9], and almost the same as in intergroup interaction (i.e., 3 vs. 3 condition). Individuals facing a three-person group (i.e., 1 vs. 3 condition) also attacked with a similar rate to individuals in interindividual interaction (i.e., 1 vs. 1 condition). On the other hand, three-person groups showed a higher attack rate when and only when they interacted with an individual.

In the previous studies, competitive intergroup interactions have been observed in the interindividual PD game [12, 13], the intergroup PD game [25, 26], and also other economic games [27–30], but no such competitiveness has been observed in some types of economic games with multiple equilibria. Wolf et al. [31] examined competitive intergroup interaction in the PD game, chicken game, battle of the sexes game, and leader game. The PD game has a unique Nash equilibrium, while the other three games have two pure-strategy Nash equilibria. In their experiments, competitive intergroup interactions were observed more frequently as compared with interindividual interactions in the PD game, but not in the other three games. These results suggest that intergroup competitiveness does not occur in games with multiple equilibria.

The payoff structure of the PS game is similar to the stag hunt game. In the cell of “attack, attack” in the strategic form of the PS game, the payoffs are determined by the first-come, first-served principle. The payoff for each party in this case of mutual attacks might be regarded as the expected payoff (i.e., 250 yen) of the cases of win (i.e., 400 yen) and loss (i.e., 100 yen), where we assume the winning probability to be 50%. If we fill this cell with this expected payoff, the game has two pure-strategy equilibria: “attack, attack” and “non-attack, non-attack.” Feri et al. [32] reported that participants did not change their choices regardless of whether they played the game as an individual with other individuals or as a member of a group with other groups in a simple 2 x 2 coordination game. Therefore, our negative result for the discontinuity effect in the PS game might be explained by the similarity in the payoff structure between the PS game and other games with multiple equilibria. The discontinuity effect in games with multiple equilibria should be investigated in future research.

The high attack rate in the 3 vs. 1 condition was also an unpredicted result. This finding might have resulted from the imbalance of the effectiveness of the attack in our experiment. If defeated, each three-person group needs to pay 1200 yen (i.e., 400 yen per member), while each individual needs to pay only 400 yen. In the 3 vs. 1 condition, therefore, the effectiveness of attack by the individual on the three-person group is three times as large as the one by the three-person group on the individual. Recent studies revealed that cooperation in the PD game increases when the benefit for full cooperation increases; conversely, cooperation decreases when the cost of cooperation increases [33]. When the cost of exiting a conflict increases, people tend to stay in the conflict and behave more selfishly [34], and the self-defense purpose readily legitimizes aggression [35]. The potential threat or fear of such an intensive attack might have induced three-person groups to commit defensive attacks when they faced an individual opponent. Alternately, the high attack rate of three-person groups against individuals might stem from three-person groups' (incorrect) expectation that individuals would be so fearful when playing against a group that individuals might attack groups more frequently. The relationship between the imbalance of attack effectiveness and the degree of fear also needs to be investigated in future research.

Finally, future research also needs to analyze the effect of time constraint on participants’ decisions to commit preemptive strikes. In the current study, participants were constrained to determine whether to push the button or not in 5 minutes. This "forced deliberation" might have influenced the results. Recently, whether intuition produces better outcomes than deliberation has been investigated in economic games [36–40]. These studies dealt with games such as the PD and the voluntary contribution to the public-good provision, in which participants are asked whether to cooperate or not, although non-cooperation is the dominant strategy. On the other hand, the PS game has two pure-strategy equilibria, and participants are asked whether to attack the other party or not. Time pressure may increase the emotional impact of fear in the PS game, and it may work differently from the PD and public-good games.

## Supporting Information

S1 Dataset Dataset of the study. (XLSX)
